# Hypoxia-induced up-regulation of VASP promotes invasiveness and metastasis of hepatocellular carcinoma: Erratum

**DOI:** 10.7150/thno.79069

**Published:** 2023-01-11

**Authors:** Zhikui Liu, Yufeng Wang, Changwei Dou, Meng Xu, Liankang Sun, Liang Wang, Bowen Yao, Qing Li, Wei Yang, Kangsheng Tu, Qingguang Liu

**Affiliations:** Department of Hepatobiliary Surgery, the First Affiliated Hospital of Xi'an Jiaotong University, 277 Yanta West Road, Xi'an 710061, China

We regret that the original version of our paper unfortunately contained some inappropriate representative images. The immunostaining images of VASP in the LV-VASP and VASP-shRNA groups in Figure 2D, and the representative images of transwell in Figures 2B, 2C, and 4B were mis-pasted when choosing representative images from the large image data. The correct versions of Figures 2 and 4 appear below. The authors confirm that these corrections made in this erratum do not affect the original conclusions. All the authors of the paper have agreed to this correction. The authors apologize for any inconvenience that the errors may have caused.

## Figures and Tables

**Figure 2:**
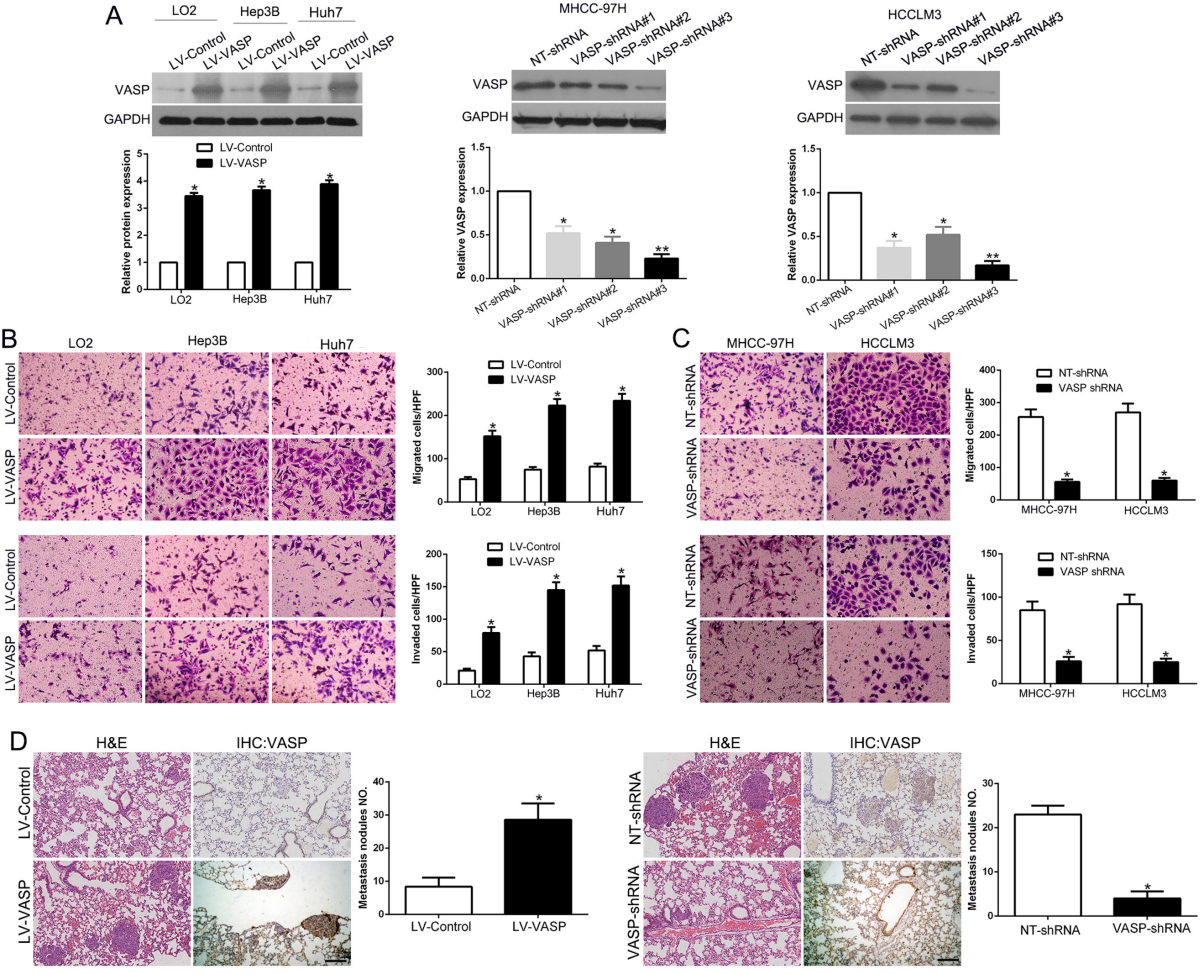
**VASP promotes HCC cell migration and invasion.** (A) Retrovirus encoding VASP vector was transduced into LO2, Hep3B, and Huh7 cells to establish stably overexpressing VASP cells. Lentivirus encoding VASP shRNA was transduced into MHCC-97H and HCCLM3 cells to establish VASP knockdown cells. (B) Comparison of the migration and invasion potential of LO2, Hep3B, and Huh7 cells transfected with LV-VASP using Transwell. (C) Comparison of migration and invasion potential of MHCC-97H and HCCLM3 cells transfected with VASP shRNA using the Transwell assay. (D) Representative hematoxylin and eosin (H&E) images of metastatic nodules from the mouse lung tissue sections of the Hep3B-VASP group (upper) and MHCC-97H shRNA group (bottom). n = six independent experiments. *P<0.05, **P<0.01.

**Figure 4:**
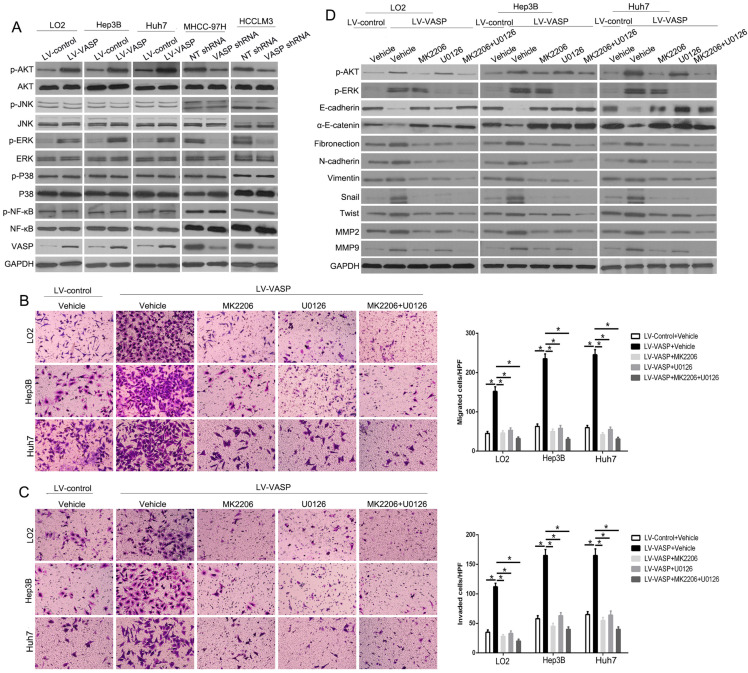
**VASP exerts oncogenic effects on HCC cells by activating AKT and ERK pathways.** (A) Western blot was performed to investigate the influence of VASP on AKT, ERK, JNK, MAPK, and NF-κB pathways in indicated cells. GAPDH was used as an internal control. (B-D) LO2, Hep3B, and Huh7 cells overexpressing VASP and corresponding cells in the control group were treated with MK2206 (AKT inhibitor) and U0126 (p-ERK inhibitor) for 24 h and subjected to (B) migration, (C) invasion, and (D) Western blotting.

